# Supplementary material for: Age-specific immunity to rotavirus infection and the risk of disease before and after rotavirus vaccine introduction in the United Kingdom: an observational, seroepidemiological study
Source: medRxiv. 2025 Apr 4:2025.04.03.25324959. Preprint. [Version 1] doi: 10.1101/2025.04.03.25324959 (PMC11998831; doi:10.1101/2025.04.03.25324959)
Supplement: Supplement 1 [file media-1.docx]

# Supplementary materials for “Age-specific immunity to rotavirus and the risk of disease before and after rotavirus vaccine introduction in the United Kingdom: an observational, seroepidemiological study”

Daniel Hungerford^1,2^, Virgina E. Pitzer^3^, Khuzwayo C. Jere^1,4^, Marc Y R Henrion^4,5^, Jonathan Mandolo^4,5^, Catherine Beavis^6^, Karen Ryan^1,2^, Jenna Lowe^1^, Nigel A. Cunliffe^1,2^, Neil French^1^, Miren Iturriza-Gómara^1,2*^

**Affiliations**

1. Department of Clinical Infection, Microbiology and Immunology, University of Liverpool, Liverpool, UK
2. NIHR Health Protection Research Unit in Gastrointestinal Infections at University of Liverpool, Liverpool, UK
3. Department of Epidemiology of Microbial Diseases, Yale School of Public Health, Yale University, New Haven, Connecticut, USA
4. Malawi-Liverpool-Wellcome Programme, Blantyre, Malawi
5. Department of Clinical Sciences, Liverpool School of Tropical Medicine, Liverpool, UK
6. NIHR public contributor, University of Liverpool, Liverpool, UK

*Current affiliation: GSK Vaccines Institute for Global Health, Siena, Italy

**Corresponding author**: Daniel Hungerford, The Ronald Ross Building, Department of Clinical Infection, Microbiology and Immunology, University of Liverpool, Liverpool, L69 7BE, UK; [d.hungerford@liverpool.ac.uk](mailto:d.hungerford@liverpool.ac.uk)

## Additional figures and tables supplementing main findings

**
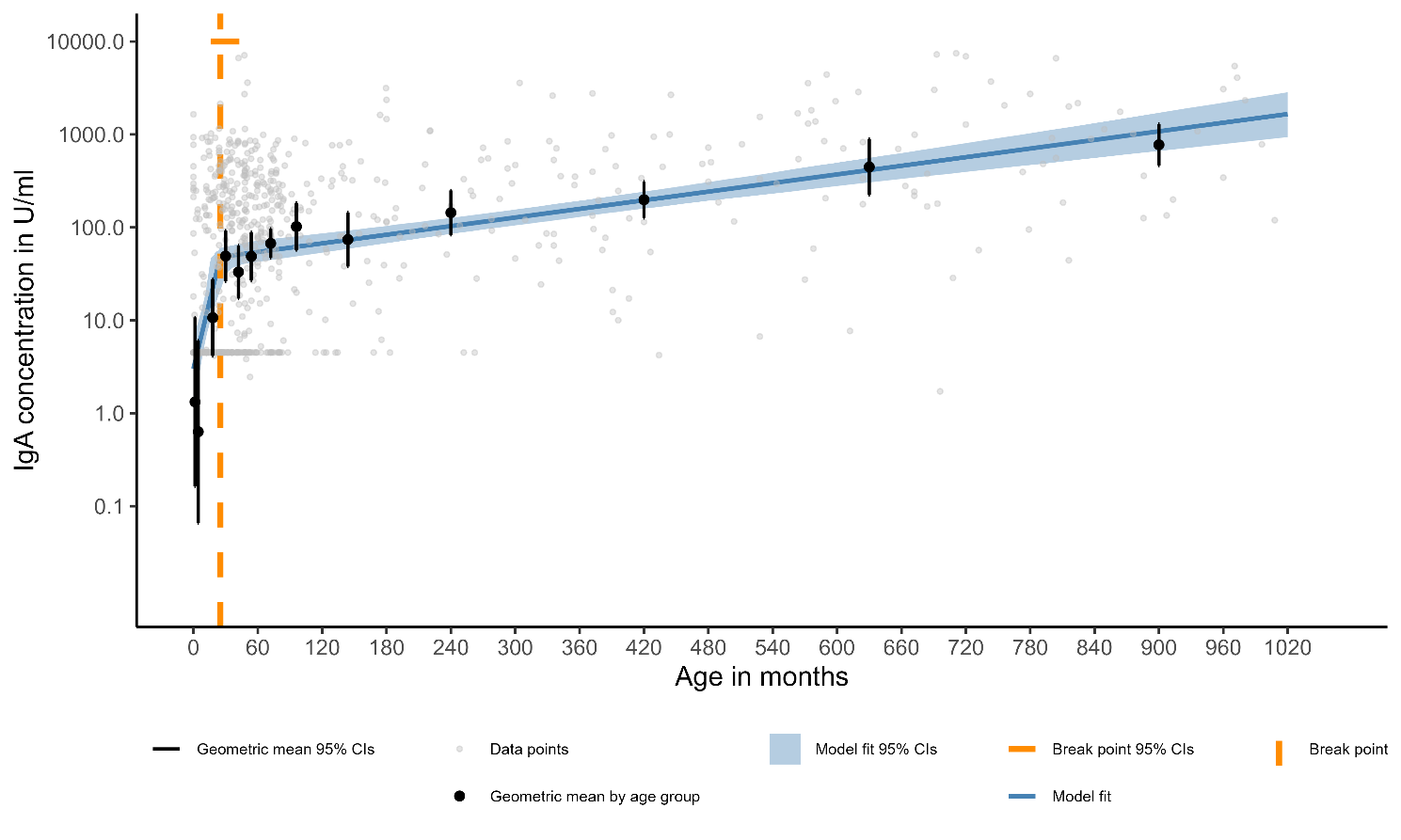
**

**Figure S1. Linear spline censored regression model assessing the associations between age and anti-rotavirus IgA for all ages in the vaccine-ineligible population**

**
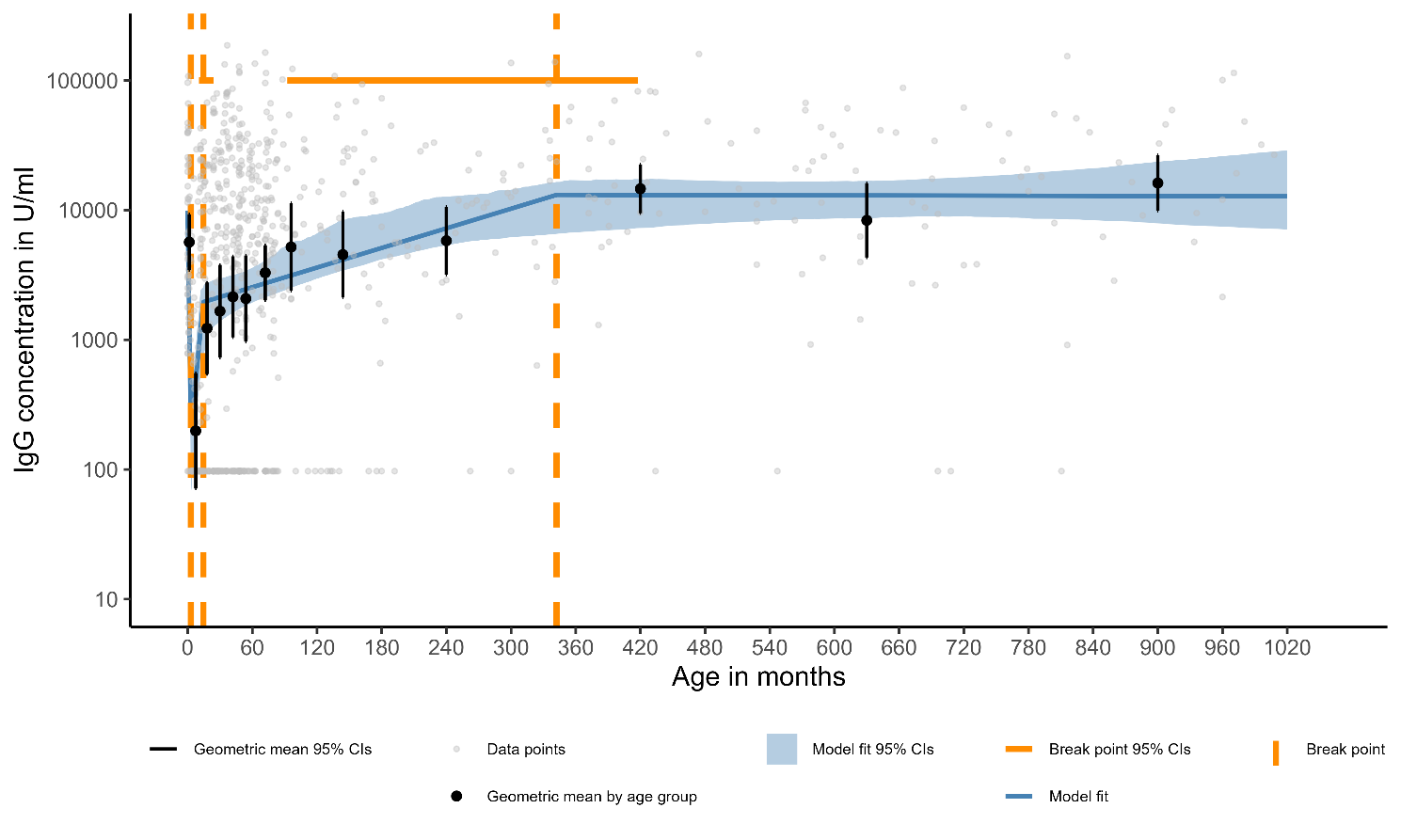
**

**Figure S2. Linear spline censored regression model assessing the associations between age and anti-rotavirus IgG for all ages in the vaccine-ineligible population**

**Table S1. Geometric mean concentrations of anti-rotavirus IgA and IgG by age group and vaccine eligibility of population**

| **Age group** | **IgA geometric mean concentration in U/ml (95% CI)** | | **IgG geometric mean concentration in U/ml (95% CI)** | |
| --- | --- | --- | --- | --- |
|  | **Vaccine-ineligible** | **Vaccine-eligible** | **Vaccine-ineligible** | **Vaccine-eligible** |
| <3m | 1.33 (0.16 to 10.74) | 0.05 (0 to 2.76) | 5665.54 (3461.42 to 9273.18) | 4411.6 (2720.88 to 7152.91) |
| 3-11m | 0.63 (0.07 to 6.05) | 18.23 (8.01 to 41.53) | 198.54 (71.31 to 552.78) | 1388.67 (675.03 to 2856.78) |
| 12-23m | 10.66 (4.14 to 27.43) | 39.58 (24.13 to 64.92) | 1226.99 (544.85 to 2763.18) | 1241.95 (568.4 to 2713.67) |
| 2y | 49.09 (26.16 to 92.1) | 37.07 (19.6 to 70.09) | 1662 (730.76 to 3779.97) | 608.23 (243.02 to 1522.31) |
| 3y | 33.08 (17.11 to 63.97) | 54.13 (32.7 to 89.62) | 2145.5 (1044.29 to 4407.94) | 1710.13 (827.12 to 3535.82) |
| 4y | 48.67 (26.76 to 88.52) | 44.61 (26.58 to 74.88) | 2080.1 (968.12 to 4469.29) | 2811.66 (1699.6 to 4651.33) |
| 5-6y | 67.18 (46.8 to 96.43) | 34.6 (20.35 to 58.83) | 3288.89 (2017.01 to 5362.79) | 1988.86 (904.58 to 4372.84) |
| 7-9y | 101.87 (57.13 to 181.65) | NA | 5195.95 (2385.48 to 11317.6) | NA |
| 10-14y | 73.88 (37.85 to 144.2) | NA | 4541.43 (2119.55 to 9730.64) | NA |
| 15-24y | 143.87 (83.02 to 249.32) | NA | 5804.99 (3185.22 to 10579.46) | NA |
| 25-44y | 198.07 (126.14 to 311.02) | NA | 14624.03 (9475.36 to 22570.35) | NA |
| 45-59y | 446.56 (223.33 to 892.91) | NA | 8344.49 (4314.49 to 16138.76) | NA |
| 60+y | 772.96 (463.2 to 1289.85) | NA | 16189.03 (9859.35 to 26582.36) | NA |

**Table S2. Proportion of IgA samples attaining correlates of protection thresholds indicative of protection against severe rotavirus disease (≥20 U/ml) and rotavirus disease of any severity (≥160 U/ml) by age group and vaccine eligibility of population**

| **Age group** | **Proportion of IgA samples ≥20 U/ml (95% CI)** | | **Proportion of IgA samples ≥160 U/ml (95% CI)** | | **Proportion of IgA samples ≥320 U/ml (95% CI)** | |
| --- | --- | --- | --- | --- | --- | --- |
|  | **Vaccine-ineligible** | **Vaccine-eligible** | **Vaccine-ineligible** | **Vaccine-eligible** | **Vaccine-ineligible** | **Vaccine-eligible** |
| <3m | 0.32 (0.2 to 0.47) | 0.15 (0.07 to 0.28) | 0.24 (0.14 to 0.39) | 0.07 (0.03 to 0.19) | 0.12 (0.05 to 0.26) | 0.05 (0.01 to 0.16) |
| 3-11m | 0.28 (0.16 to 0.43) | 0.54 (0.38 to 0.7) | 0.1 (0.04 to 0.23) | 0.23 (0.12 to 0.39) | 0.05 (0.01 to 0.17) | 0.09 (0.03 to 0.22) |
| 12-23m | 0.46 (0.34 to 0.58) | 0.77 (0.65 to 0.86) | 0.32 (0.22 to 0.44) | 0.2 (0.12 to 0.32) | 0.16 (0.09 to 0.27) | 0.15 (0.08 to 0.26) |
| 2y | 0.69 (0.58 to 0.79) | 0.64 (0.5 to 0.76) | 0.47 (0.36 to 0.58) | 0.3 (0.19 to 0.44) | 0.29 (0.2 to 0.4) | 0.14 (0.07 to 0.26) |
| 3y | 0.61 (0.5 to 0.72) | 0.76 (0.63 to 0.86) | 0.32 (0.22 to 0.43) | 0.39 (0.27 to 0.53) | 0.21 (0.13 to 0.32) | 0.12 (0.06 to 0.23) |
| 4y | 0.69 (0.58 to 0.78) | 0.69 (0.57 to 0.79) | 0.42 (0.31 to 0.53) | 0.31 (0.21 to 0.43) | 0.3 (0.21 to 0.41) | 0.18 (0.1 to 0.29) |
| 5-6y | 0.8 (0.71 to 0.87) | 0.67 (0.5 to 0.8) | 0.4 (0.31 to 0.5) | 0.18 (0.09 to 0.34) | 0.15 (0.09 to 0.24) | 0.03 (0.01 to 0.15) |
| 7-9y | 0.85 (0.66 to 0.94) | NA | 0.42 (0.26 to 0.61) | NA | 0.19 (0.09 to 0.38) | NA |
| 10-14y | 0.76 (0.6 to 0.87) | NA | 0.38 (0.24 to 0.54) | NA | 0.32 (0.2 to 0.49) | NA |
| 15-24y | 0.91 (0.78 to 0.97) | NA | 0.51 (0.36 to 0.67) | NA | 0.31 (0.19 to 0.48) | NA |
| 25-44y | 0.9 (0.79 to 0.96) | NA | 0.57 (0.43 to 0.69) | NA | 0.47 (0.34 to 0.6) | NA |
| 45-59y | 0.94 (0.8 to 0.98) | NA | 0.84 (0.68 to 0.93) | NA | 0.59 (0.42 to 0.74) | NA |
| 60+ | 1 (0.89 to 1) | NA | 0.81 (0.65 to 0.91) | NA | 0.75 (0.58 to 0.87) | NA |

## Sensitivity analyses described in the main paper

**Table S3. Association between vaccine eligibility and the proportion of IgA samples attaining correlates of protection thresholds indicative of protection against severe rotavirus disease (≥20 U/ml) and rotavirus disease of any severity (≥160 U/ml and (≥320 U/ml) in children under the age of 7 years**

| **Analysis** | **CoP IgA ≥20 U/ml** | | | **CoP IgA ≥160 U/ml** | | | **CoP IgA ≥320 U/ml** | | |
| --- | --- | --- | --- | --- | --- | --- | --- | --- | --- |
|  | **Vaccine-ineligible**  **(%)** | **Vaccine-eligible**  **(%)** | **aOR**  **(95% CI)** | **Vaccine-ineligible**  **(%)** | **Vaccine-eligible**  **(%)** | **aOR**  **(95% CI)** | **Vaccine-ineligible**  **(%)** | **Vaccine-eligible**  **(%)** | **aOR**  **(95% CI)** |
| Original | 279/464 (60.1) | 207/332 (62.3) | 1.29 (0.95 to 1.75) | 163/464 (35.1) | 83/332 (0.25) | 0.66 (0.48 to 0.91) | 91/464 (19.6) | 39/332 (11.7) | 0.56 (0.37 to 0.84) |
| Sensitivity* | 188/322 (58.4) | 207/332 (62.3) | 1.23 (0.89 to 1.71) | 108/322 (33.5) | 83/332 (0.25) | 0.67 (0.48 to 0.94) | 59/322 (18.3) | 39/332 (11.7) | 0.60 (0.38 to 0.92) |

*(vaccine-ineligible population only includes those with a sample collected in the pre-vaccine era)


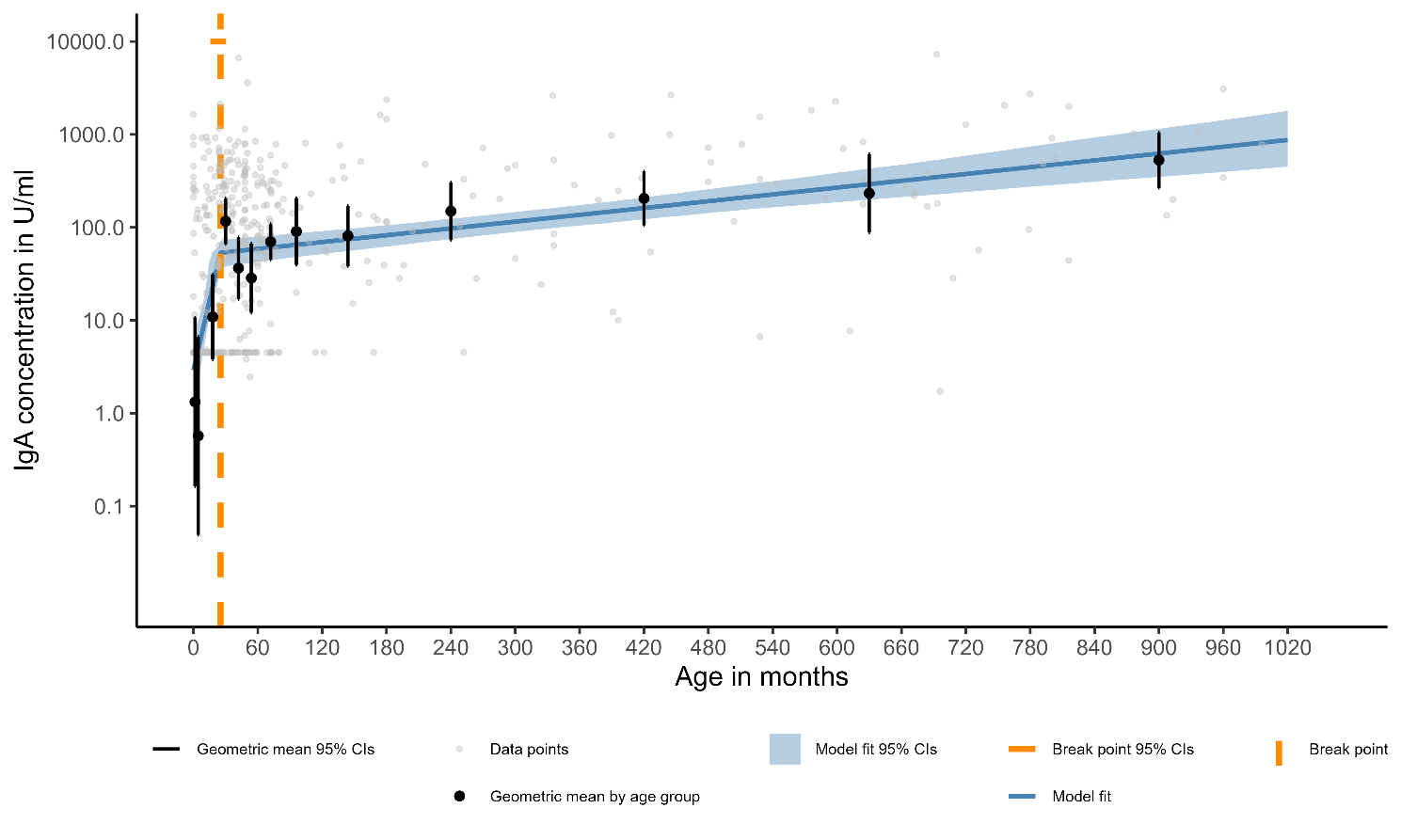


**Figure S3. Linear spline censored regression model assessing the associations between age and anti-rotavirus IgA for all ages in the vaccine-ineligible, with sample collected in the pre-vaccine era only**

**
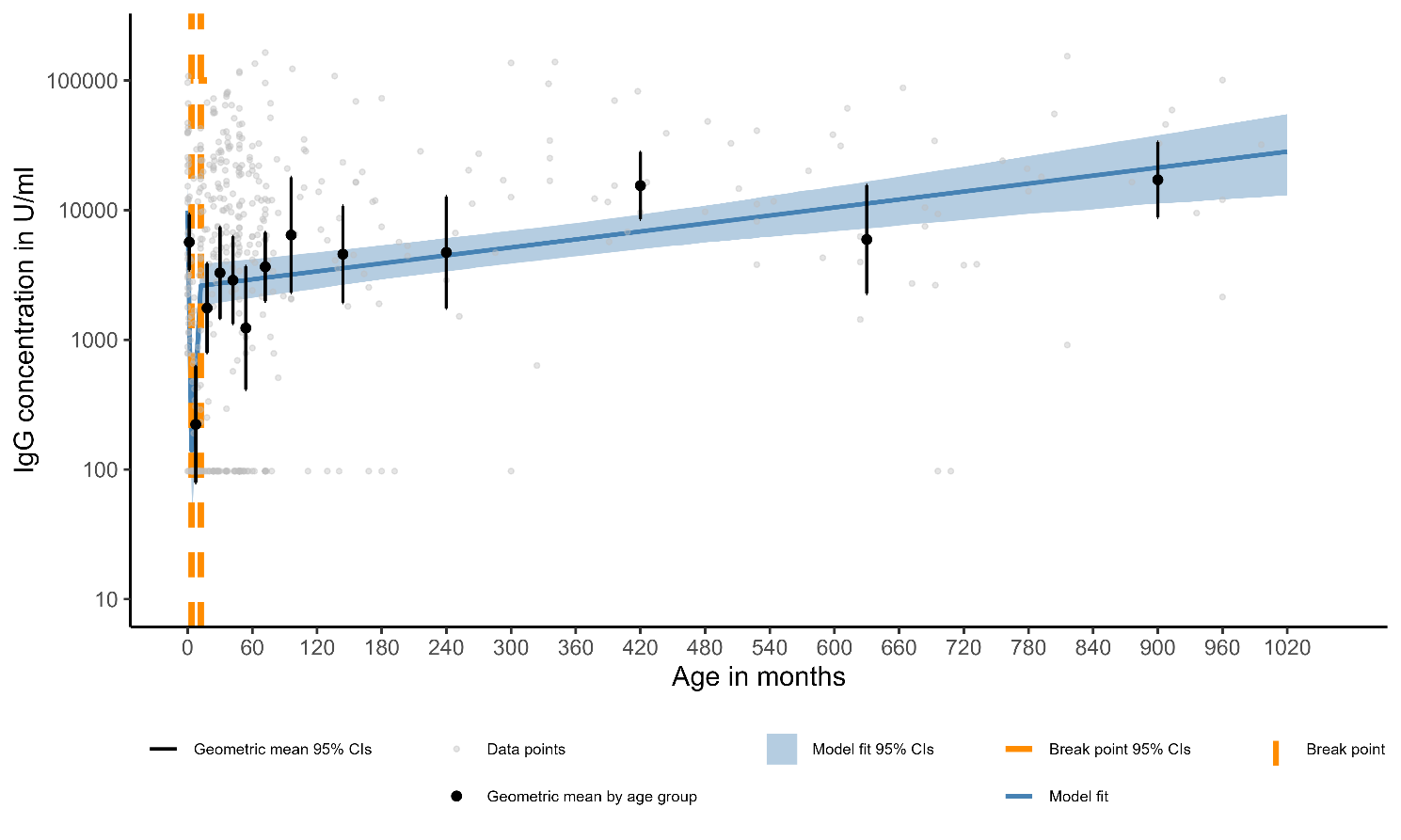
Figure S4. Linear spline censored regression model assessing the associations between age and anti-rotavirus IgG for all ages in the vaccine-ineligible, with sample collected in the pre-vaccine era only**

**Table S4. Knot and slope parameter estimates from linear spline censored regression for anti-rotavirus IgA and IgG in all vaccine-ineligible compared to models which only include samples from vaccine-ineligible collected in pre-vaccine era**

| Vaccine ineligibility | Knots in age in months (95% CI) | | | Model slopes ^ⴕ^ in log(U/ml)/year (95% CI) | | | |
| --- | --- | --- | --- | --- | --- | --- | --- |
|  | Knot 1 | Knot 2 | Knot 3 | Slope 1 | Slope 2 | Slope 3 | Slope 4 |
| IgA | | | | | | | |
| All samples | 24.96 (16.08 to 42.82) |  | - | 1.33 (0.67 to 2.63) | 0.05 (0.04 to 0.05) |  |  |
| Sample collected in pre-vaccine era only | 25.20 (15.71 to 30.48) |  |  | 1.38 (0.90 to 2.53) | 0.04 (0.02 to 0.05) |  |  |
| IgG | | | | | | | |
| All samples | 3.00 (2.82 to 3.38) | 14.56 (10.52 to 24.00) | 342.33 (92.44 to 417.61) | -15.48 (-18.96 to -13.32) | 2.34 (1.28 to 4.55) | 0.07 (0.05 to 0.25) | 0.00 (-0.01 to 0.02) |
| Sample collected in pre-vaccine era only | 3.60 (2.74 to 5.30) | 12.17 (10.07 to 18.00) |  | -14.28 (-17.76 to -9.01) | 4.13 (1.96 to 7.37) | 0.02 (0.01 to 0.04) |  |

red text = positive slope; blue text = negative slope; black text = null slope

^ⴕ^ slopes (m) are from a log-linear model $y=c^{e(\mathrm{mx})}$, m therefore takes the units which are the inverse of the units of x-axis, c = intercept, x = value of x (age in months), y = value of y (concentration in U/ml)
